# Transcriptomic Analysis Reveals Mechanisms of Sterile and Fertile Flower Differentiation and Development in *Viburnum macrocephalum* f. *keteleeri*

**DOI:** 10.3389/fpls.2017.00261

**Published:** 2017-03-01

**Authors:** Zhaogeng Lu, Jing Xu, Weixing Li, Li Zhang, Jiawen Cui, Qingsong He, Li Wang, Biao Jin

**Affiliations:** College of Horticulture and Plant Protection, Yangzhou UniversityYangzhou, China

**Keywords:** sterile flower, fertile flower, transcriptome, gene expression, differentiation and development, *Viburnum macrocephalum* f. *keteleeri*

## Abstract

Sterile and fertile flowers are an important evolutionary developmental (evo-devo) phenotype in angiosperm flowers, playing important roles in pollinator attraction and sexual reproductive success. However, the gene regulatory mechanisms underlying fertile and sterile flower differentiation and development remain largely unknown. *Viburnum macrocephalum* f. *keteleeri*, which possesses fertile and sterile flowers in a single inflorescence, is a useful candidate species for investigating the regulatory networks in differentiation and development. We developed a *de novo*-assembled flower reference transcriptome. Using RNA sequencing (RNA-seq), we compared the expression patterns of fertile and sterile flowers isolated from the same inflorescence over its rapid developmental stages. The flower reference transcriptome consisted of 105,683 non-redundant transcripts, of which 5,675 transcripts showed significant differential expression between fertile and sterile flowers. Combined with morphological and cytological changes between fertile and sterile flowers, we identified expression changes of many genes potentially involved in reproductive processes, phytohormone signaling, and cell proliferation and expansion using RNA-seq and qRT-PCR. In particular, many transcription factors (TFs), including MADS-box family members and ABCDE-class genes, were identified, and expression changes in TFs involved in multiple functions were analyzed and highlighted to determine their roles in regulating fertile and sterile flower differentiation and development. Our large-scale transcriptional analysis of fertile and sterile flowers revealed the dynamics of transcriptional networks and potentially key components in regulating differentiation and development of fertile and sterile flowers in *Viburnum macrocephalum* f. *keteleeri*. Our data provide a useful resource for *Viburnum* transcriptional research and offer insights into gene regulation of differentiation of diverse evo-devo processes in flowers.

## Introduction

Flower development is attracting great attention as a fascinating topic for studying plant development and evolution. Angiosperm flowers and inflorescences display great diversity in morphology, with various shapes, sizes, and other traits (Cooley et al., 2008), underlying the diverse consequences of the evolutionary development (“evo-devo”) of flowering plants. According to the capacity for sexual reproduction and gamete formation, flowers can be divided into fertile and sterile flowers. Fertile flowers are capable of producing fertile gametes for further generations, due to their normal sexual organs. In contrast, sterile flowers have abnormal stamens, defective anthers, or no viable pollen, and thus fail to produce seeds (Donoghue et al., 2003; Jin et al., 2010). Many sterile flowers are far larger and more conspicuous than fertile flowers within the same inflorescence (Nielsen et al., 2002; Donoghue et al., 2003; Jin et al., 2010). Such sterile flowers exist in many genera, including *Viburnum* (Adoxaceae) and *Hydrangea* (Hydrangeaceae), and in the Asteraceae family, and are considered to be an evolutionary consequence of long-term ecological selection by pollinator attraction, which plays an important role in enhancing reproductive success (Donoghue et al., 2003; Jin et al., 2010). However, the developmental regulation of sterile flowers, which makes them conspicuously different from fertile flowers in appearance and structure, remains unclear.

RNA-seq approaches have been used extensively to characterize gene expression and determine genetic networks in flower development (Ó'Maoiléidigh et al., 2014; Zhang et al., 2014). In recent years, many of the key floral regulators in *Arabidopsis thaliana* and other species have been identified through large-scale analyses of floral transcriptomes (Ó'Maoiléidigh et al., 2014; Zhang et al., 2014; Vining et al., 2015). For example, the MADS-box family genes encode a family of transcription factors that control diverse developmental processes such as flowering time, meristem identity, and floral organ identity (Becker and Theißen, 2003; Ó'Maoiléidigh et al., 2014). The ABCDE-class genes act in a combinatorial way to specify sepal, petal, stamen, carpel, and ovule formation (Pelaz et al., 2000; Theissen and Melzer, 2007). Many other genes, including genes encoding transcription factors (TFs), have also been shown to be required for the development of anthers, pollen, and the tapetum. For instance, the altered function of *ABORTED MICROSPORES* (*AMS*; Xu et al., 2010), callose synthase 5 (*CALS5*; Dong et al., 2005), SBP-Like 8 (*SPL8*; Xing et al., 2010), or *EXCESS MICROSPOROCYTES1/EXTRA SPOROGENOUS* (*EMS1*/*EXS*; Canales et al., 2002) can result in reduced fertility or male sterility in flowering plants. Phytohormone signaling molecules, including auxin (Cecchetti et al., 2008), gibberellin (Cheng et al., 2004), jasmonate (Yuan and Zhang, 2015), cytokinin (Bartrina et al., 2011; Han et al., 2014), and brassinosteroid (Ye et al., 2010), are involved in regulating the development and fertility of flowers. For example, gibberellins promote flower growth via cell expansion and/or proliferation (Achard et al., 2009). Overexpression of jasmonate signaling pathway proteins (JAZs) usually results in low fertility or male sterility (Yuan and Zhang, 2015). These investigations have indicated the presence of a complex gene regulatory network underlying floral organ development and fertility; however, our current knowledge and understanding of the gene regulatory networks involved in the differentiation and development of sterile and fertile flowers remain limited.

**Figure 1:**
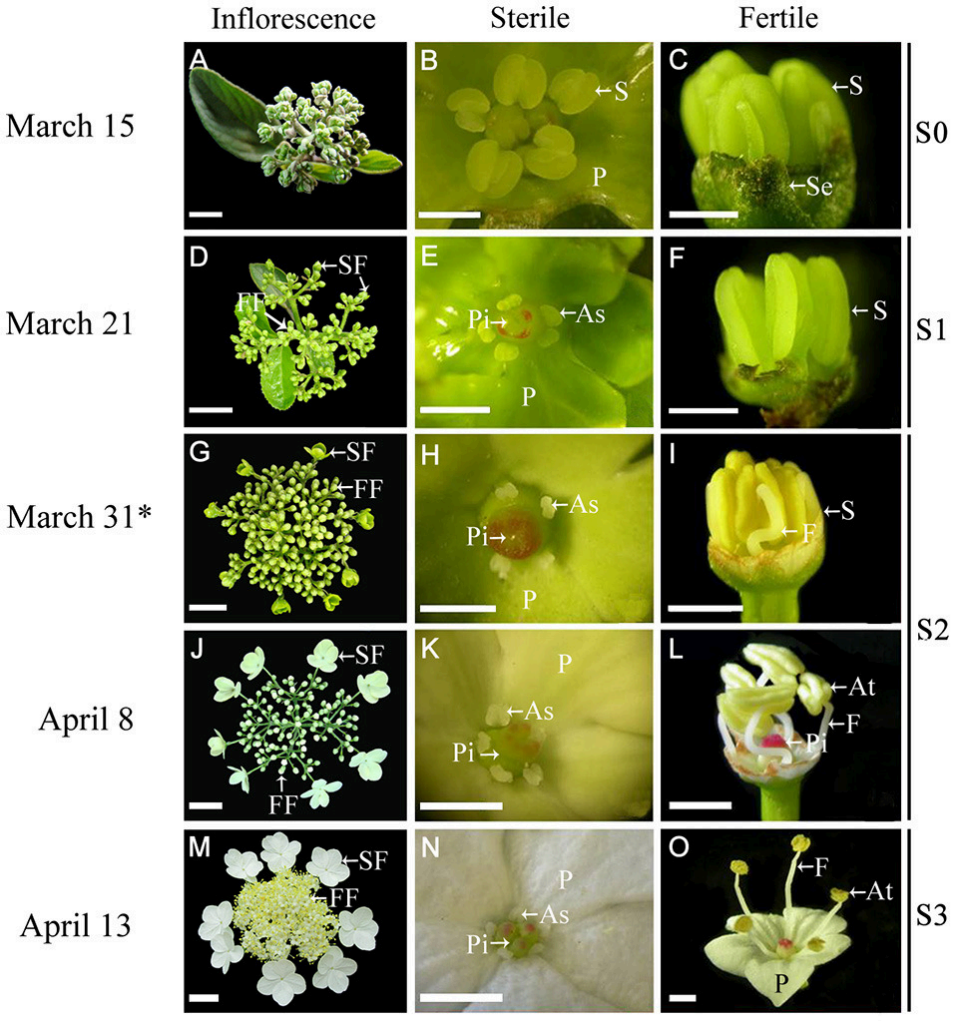
**Morphological comparison of fertile and sterile flowers during development stages in ***V. macrocephalum*** f. ***keteleeri***. (A–C)** Inflorescence at early developmental stages (March 15, S0). Normal stamens, pistils, and petals were seen in fertile and sterile flowers. **(D–F)** Inflorescence ~1 week before anthesis (March 21, S1). Stamens (or anthers) and pistils (or stigmas) of sterile flowers appeared abnormal, but were normal in fertile flowers. **(G–L)** Inflorescence at the rapid developmental stage (March 31 to April 8, at anthesis, S2). Sterile flowers generally bloomed, with enlarged petals and degraded stamens and pistils, whereas fertile flowers showed elongated filaments, plump anthers, and pistils. **(M–O)** Inflorescence at the peak flowering stage (April 13, S3). Fully degraded stamens and pistils were seen in sterile flowers, whereas dehiscent anthers produced pollen grains in fully developed fertile flowers. SF, sterile flower; FF, fertile flower; S, stamen; P, petal; Pi, pistil; As, abnormal stamen; At, anther; Se, sepal; and F, filament. Asterisks indicate RNA-seq samples for Illumina sequencing. Bars = 2 cm **(A)**, 3 cm **(D,G,J,M)**, 1 mm **(B,C,E,F,H,K,N)**, 2 mm **(I,L,O)**.

*Viburnum macrocephalum* f. *keteleeri*, a Chinese wild shrub, is a useful candidate species for investigating sterile and fertile flowers (Jin et al., 2010). Its inflorescence consists of an outer ring of eight large sterile flowers surrounding a center of small bisexual fertile flowers (Figure 1M). Previous morphological and anatomical studies in this species have shown that sterile and fertile flowers are similar during the early developmental stages and diverge in subsequent developmental stages (Jin et al., 2010). The divergence between sterile and fertile flowers is prominent in the blooming stage. Relative to normal fertile flowers, sterile flowers have big, showy petals, ruptured stigmas, defective anthers, and abnormal microsporogenesis, apparently with a role in pollinator attraction (Jin et al., 2010). These findings showed that the sterile flowers developed and differentiated from early fertile flowers in the same inflorescence, and thus could be model materials to compare the formation mechanism with that of fertile flowers in the same genetic background.

To investigate the gene and molecular regulation mechanism underlying the development of fertile and sterile flowers, we used Illumina RNA-seq technology to generate a comprehensive floral transcriptome from *V. macrocephalum* f. *keteleeri*. Combined with morphological and cytological comparisons between fertile and sterile flowers, we screened and identified candidate differentially expressed genes (DEGs). A global analysis of TFs was performed to identify differentially expressed TFs. We also performed quantitative reverse transcription PCR (qRT-PCR) experiments to determine expression changes in several key regulators involved in multiple functions at different developmental stages. These results provide a first comprehensive overview of the genes and related functions that are required for the differentiation and development of sterile and fertile flowers.

## Materials and methods

### Plant materials and RNA extraction

Fertile and sterile flowers were collected from 15-year-old *V. macrocephalum* f. *keteleeri* plants grown on the campus of Yangzhou University (32°39′ N, 119°43′ E, Yangzhou, China) under natural conditions. To collect samples for RNA, fertile and sterile flowers from inflorescences at various developmental stages [early developmental stage S0 (Figures 1A–C), initial flowering stage S1 (Figures 1D–F), rapid flowering stage S2 (Figures 1G–L), and peak flowering stage S3 (Figures 1M–O)] were sampled separately, snap-frozen in liquid nitrogen, and stored at −80°C until used for total RNA isolation. The fertile and sterile flowers from earlier developmental stages (S0, S1) are difficult to distinguish by morphological observation, although their anatomical structural differences can be visualized under a stereomicroscope in S1. Thus, fertile and sterile flowers derived from one inflorescence at S2 (March 31) were selected for RNA-seq. Three biological replicates for each sample were selected randomly from three individuals, and each biological replicate contained 4–6 sterile or fertile flowers. Samples from fertile and sterile flowers at S1, S2, and S3 were used for qRT-PCR experiments. Additionally, 10 inflorescences with fertile and sterile flowers were collected and prepared for morphological and anatomical observations.

All total RNA samples were extracted from fertile and sterile flowers using the Mini BEST Plant RNA Extraction Kit (TaKaRa, Dalian, China) and treated with genomic DNA (gDNA) Eraser (TaKaRa, Dalian, China) to reduce or eliminate any DNA contamination. RNA quality and quantity were determined using a Nanophotometer spectrophotometer (IMPLEN, CA, USA) and the Qubit RNA Assay Kit with a Qubit 2.0 Fluorometer (Life Technologies, CA, USA). RNA integrity was assessed using the RNA Nano 6000 Assay Kit for the Agilent Bioanalyzer 2100 system (Agilent Technologies, CA, USA), and RNA samples with RNA integrity numbers (RINs) > 7.1 were used for RNA-seq.

### Morphological and anatomical observations

For inflorescences containing fertile and sterile flowers at different developmental stages, we first took photographs against a black background using a digital camera. Similarly, the developmental processes of stamens and pistils within fertile and sterile flowers were captured using a stereomicroscope (Olympus SZX7, Tokyo, Japan). In addition, petal lengths and widths of fertile and sterile flowers were determined using AutoCAD software, based on photographs from 30 samples at the S1, S2, and S3 stages.

From morphological observations, about 20 petal specimens were cut separately from fertile and sterile flowers at S2, using a razor blade, and ~3 mm^3^ of each sample was prefixed in 2.5% (v/v) glutaraldehyde (in 0.1 mol/L phosphate buffer, pH 7.2) at 4°C overnight. After postfixing in 1% (w/v) osmium tetroxide for 6 h at room temperature, the samples were washed three times in 0.2 M phosphate buffer (pH 7.2), dehydrated through an ethanol series, treated twice for 30 min with propylene oxide, and then infiltrated with 1:1 propylene oxide/resin in embedding capsules overnight, before finally embedding in Spurr's resin (Wang et al., 2016). For ultrastructural observations, 70 nm-thick sections were cut with a Leica EM UC6 ultramicrotome (Leica Microsystems GmbH, Wetzlar, Germany), and stained with 1% (w/v) uranyl acetate and 1% (w/v) lead citrate. Petals cells were observed and photographed under a Philips Tecnai 12 transmission electron microscope (JEOL Ltd., Tokyo, Japan).

### Illumina sequencing and *de novo* assembly

RNA (~3 μg per sample) was used as the input material for constructing libraries. RNA-seq libraries were prepared using the TruSeq Paired-End (PE) Cluster Kit v3-cBot-HS (Illumina, PE125) according to the manufacturer's protocol. Libraries from fertile and sterile flowers, with three biological replicates, were sequenced in a single Illumina Hiseq 2500 flowcell, generating >139 million paired-end reads per sample. A Perl script was written to remove low-quality sequences (reads with a base quality < 20). For *de novo* reference transcriptome assembly, all high-quality RNA-Seq reads were pooled from the Illumina sequencing of each of the six samples (three biological replicates) and were then used as input for assembly using Trinity software (Grabherr et al., 2011). All raw sequence data have been deposited in the NCBI Sequence Read Archive (SRA, accession number SRP076665).

### Functional annotation and classification

All Illumina-assembled unigenes (the longest transcript for each gene) were aligned against the NCBI non-redundant protein (Nr) (http://www.ncbi.nlm.nih.gov/), NCBI non-redundant nucleotide sequence (Nt), Pfam (http://pfam.xfam.org/), KOG (http://www.ncbi.nlm.nih.gov/COG/), Swiss-Prot (http://www.uniprot.org/), and KEGG (http://www.genome.jp/kegg) databases using BLASTX alignments with an *E*-value cut-off of 10^−5^. With Nr annotation, Gene ontology (GO) annotations of unigenes were obtained using the Blast2GO software (http://www.geneontology.org; Götz et al., 2008). GO has three ontologies describing molecular function, cellular components, and biological processes (Ashburner et al., 2000). We then used the WEGO software to perform GO functional classifications of all unigenes to understand the distribution of gene functions at the macro level (Ye et al., 2006). Based on KEGG mapping, unigenes were assigned to multiple pathways, using BLASTx, thereby retrieving KEGG Orthology (KO) information.

### Differential gene expression analysis

Before performing differential expression analysis of unigenes, we estimated gene expression levels for each sample using the RSEM software package (Li and Dewey, 2011). The FPKM (expected number of fragments per kilobase of transcript sequence per million base pairs sequenced) value was used to quantify gene expression levels (Trapnell et al., 2010), which takes the influence of both the sequencing depth and gene length on read count into account. These expressed data sets are available at the NCBI GEO, under accession number GSE83429. Next, we conducted a differential expression analysis of two conditions using the DESeq R package (ver. 1.10.1; Anders and Huber, 2010). DESeq provides statistical routines for determining differential expression in digital gene expression data using a model based on a negative binomial distribution. The *P*-value was adjusted using the Benjamini and Hochberg approach (Benjamini and Hochberg, 1995). Genes with an adjusted *P*-value < 0.05, as found by DESeq, were deemed to be differentially expressed. GO functional enrichment analysis of the differentially expressed genes (DEGs) was carried out with the GOseq R package, based on a Wallenius non-central hyper-geometric distribution (Young et al., 2010), which can find significantly enriched GO terms in DEGs vs. the genome background. To understand high-level functions and utilities of the biological system, all DEGs were assigned to the diverse pathways of the KEGG database. Then, we used the KOBAS software to test the statistical enrichment of differentially expressed genes within the KEGG pathways (Mao et al., 2005).

### qRT-PCR validation and expression analysis

We conducted qRT-PCR experiments to confirm and analyze basic expression levels of a subset of candidate functional genes. Treated RNA solutions (10 μL) (without DNA contamination) from fertile and sterile flowers at S1, S2, and S3 were subjected to reverse transcriptase reactions with the PrimeScript RT Reagent Kit (TaKaRa, Dalian, China) according to the manufacturer's protocol. Gene-specific primers were designed using Primer 5.0 software (Table S1). The *SAND* (NC_003071.7) gene was used as a housekeeping gene to normalize the expression of the investigated genes. qRT-PCR was performed using a CFX Connect Real-Time thermal cycler (Bio-Rad, USA) using a SYBR Premix Ex Taq Kit (TaKaRa) following the manufacturer's protocol. PCR reactions were performed as follows: 95°C for 30 s, followed by 40 cycles of 95°C for 5 s, 60°C for 30 s, and 72°C for 10 s. Each reaction had three biological replicates, and comparative threshold (Ct) values were determined with the Bio-Rad CFX Manager software (ver. 3.1.1517.0823). Relative expression levels of target genes were calculated using the 2^−ΔΔ*Ct*^ method (Livak and Schmittgen, 2001). Standard errors of the mean among the replicates were calculated. Non-overlapping letters (a–c) indicate significant differences between fertile or sterile flowers at different stages, based on ANOVA analysis and Multiple Range Tests with a confidence level of 95%. Similar significance analyses were conducted comparing fertile and sterile flowers in each stage.

### Phylogenetic analyses

The MADS-box gene sequences used were from the *V. macrocephalum* f. *keteleeri* transcriptome and from *A. thaliana*. The *A. thaliana* MADS-box gene sequences were downloaded from the Arabidopsis Information Resource (TAIR10) (http://www.arabidopsis.org). All multiple sequence alignments and phylogenetic trees for MADS-box genes were constructed using MEGA6.06 software and the neighbor-joining (NJ) algorithm, according to the manual (Tamura et al., 2013). Bootstrap analyses with 1,000 replicates were used to assess the robustness of the tree.

## Results

### *De novo* assembly of the *V. macrocephalum* f. *keteleeri* flower transcriptome

We observed inflorescence development at four stages, from March 15 to April 13 (Figure 1). At the early stage (March 15, S0) sterile flowers developed five petals and one pistil surrounded by five stamens, similar to fertile flowers (Figures 1A–C). One week later (March 21, before anthesis, S1), the fertile flowers had normal stamens (or anthers) and pistils (Figures 1D,F), while the sterile flowers exhibited degenerated stamens (or anthers) and ruptured stigmas (Figures 1D,E). During the rapid developmental process (March 31 to April 8, at anthesis, S2), sterile flowers bloomed gradually, petals enlarged (Figures 1G,J), and stamens and pistils continued to deform or collapse (Figures 1H,K), whereas the fertile flowers developed elongated filaments, plump anthers, and pistils (Figures 1I,L). At the peak flowering stage (April 13, S3), fertile flowers produced pollen grains, and pistils were developed fully (Figures 1M,O), whereas in the sterile flowers, the stamens and pistils had degenerated completely (Figure 1N).

**Figure 2:**
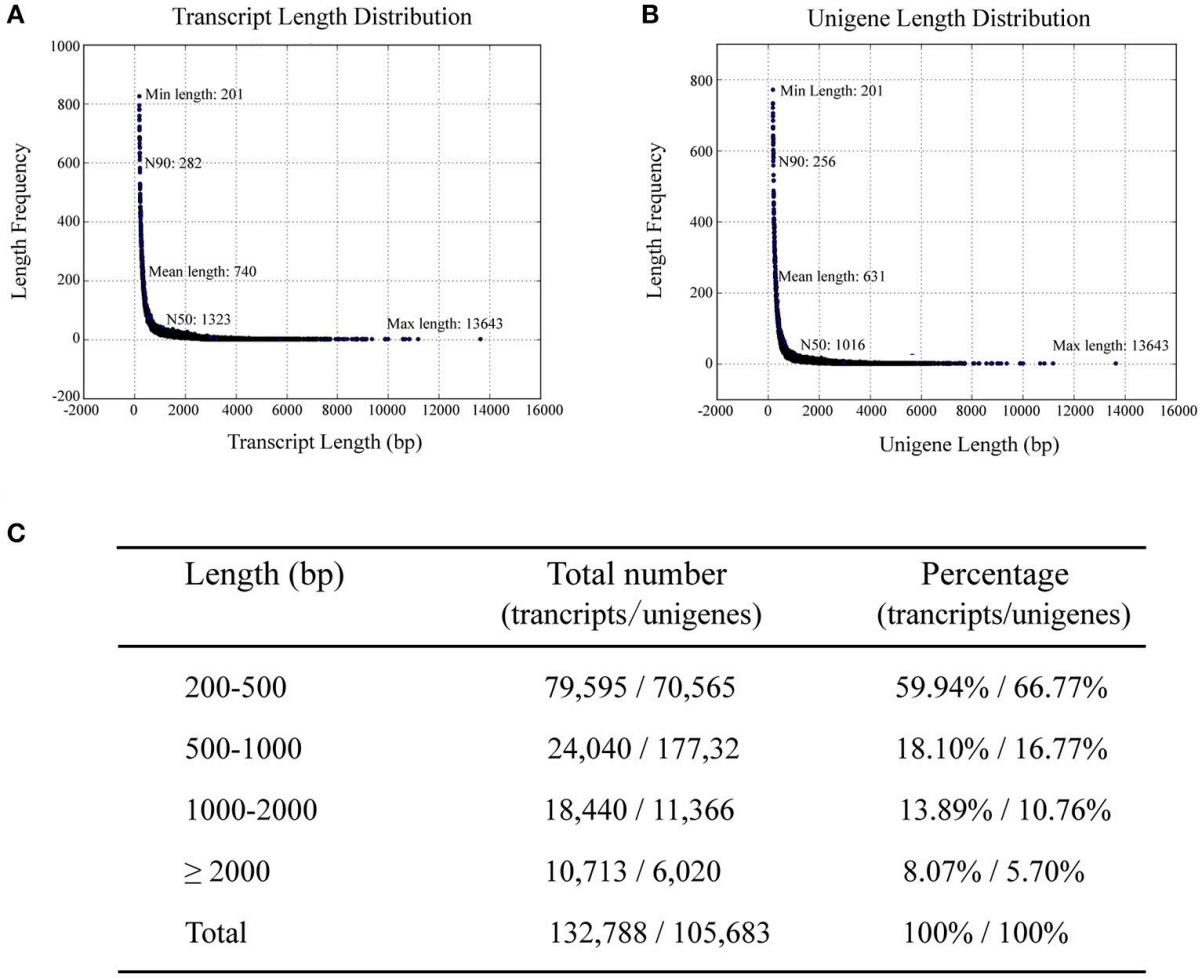
**Length distribution of assembled transcripts and unigenes. N50/N90 represents 50 or 90% length of all unigenes sequences. (A)** Distribution of assembled transcripts. **(B)** Distribution of assembled unigenes. **(C)** Percentage of assembled transcripts and unigenes within different length intervals.

To investigate differences in the transcriptomes of sterile and fertile flowers, we sequenced RNA samples extracted from sterile and fertile flowers at S2 (March 31), using the Illumina Hiseq2500 platform. Three biological replicates were prepared for sterile and fertile flowers, resulting in 139.05 and 139.86 million raw reads in the two samples, respectively (Table S2). After removal of filtering adapters, low-quality sequences, and ambiguous reads, we obtained approximately 133.40 million and 134.32 million paired-end clean reads in sterile and fertile flowers, respectively (Table S2). In total, 267.72 million pooled clean reads were used for the assembly of sequences with the *de novo* Trinity software. This assembly resulted in 132,788 transcripts with a mean length of 740 bp (N50 = 1323 bp; Figure 2A), and included 105,683 unigene sequences with a mean length of 631 bp (N50 = 1016 bp; Figure 2B). Sequences ranging from 200 to 2,000 bp in length accounted for nearly 91.9% of the total transcripts and 94.3% of the total unigenes. In total, 10,713 (8.1%) transcripts and 6020 (5.7%) unigenes were >2,000 bp in length (Figure 2C). For the validation and annotation of the assembled unigenes, all unigene sequences (105,683 unigenes) were searched against public protein databases using the BlastX program (*E* < 1e^−5^). The results indicated that 20,724 (19.6%) unigenes had significant matches in the Nt database, while 38,224 (36.16%), 26,744 (25.3%), 26,629 (25.19%), 13,637 (12.9%), 30,381 (28.74%), and 12201 (11.54%) unigenes showed significant similarities to known proteins in the Nr, Swiss-Prot, Pfam, KOG, GO, and KO databases, respectively (Figure S1A). Of the 105,683 unigenes, 43,870 (41.51%) were successfully annotated from at least one database. Additionally, the species distribution in the Nr database showed that 18,615 (48.70%) unigenes had highest similarities to sequences from *Vitis vinifera* (48.7%), *Populus trichocarpa* (10.60%), or *Ricinus communis* (9.20%; Figure S1B).

To further characterize the functional classifications of the annotated unigenes, we searched the annotated sequences for genes involved in GO classifications. Using Nr annotations, 30,381 (28.74%) unigenes could be categorized into 58 functional groups and summarized into the three main GO categories (biological processes, cellular components, and molecular functions; Figure S1C). In each of the three main GO classifications, “binding,” “cell,” and “cellular process” were the most highly represented groups. We also noticed some identified genes involved in other important biological processes, such as reproductive processes and growth. KEGG analysis revealed the biological pathways in which the unigenes were likely involved. Assembled unigenes were compared with the KEGG database using BLASTx and the corresponding pathways were identified. In total, 12,201 unigenes showed significant matches and were assigned to 274 KEGG pathways (Figure S1D). A large proportion of these unigenes belonged to translation (1,530 unigenes), followed by signal transduction (1,254 unigenes), and carbohydrate metabolism (1,169 unigenes). We also noticed that many genes were involved in cell growth and death (428 unigenes) and developmental (75 unigenes) pathways.

### Global analyses of gene expression profiles and distinct enrichment analysis of DEGs between sterile and fertile flowers

**Figure 3:**
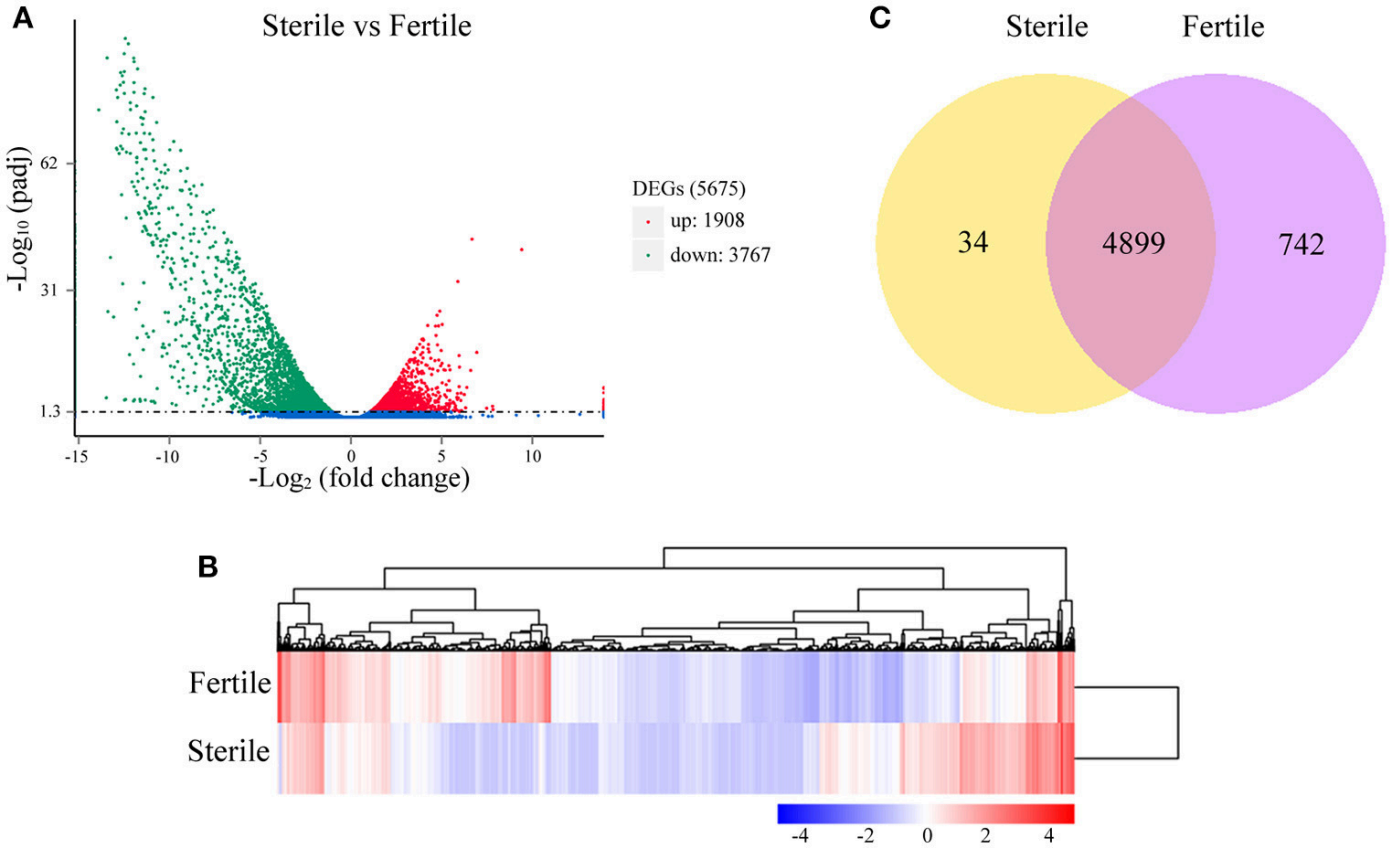
**Distribution of DEGs between fertile and sterile flowers. (A)** Red spots represent upregulated DEGs and green spots indicate downregulated DEGs. Those shown in blue are unigenes that did not show obvious changes. **(B)** Clustering analysis of all DEGs between fertile and sterile flowers. Each row corresponds to a gene, while the samples are represented by the columns. The expression levels for each gene [(log^10^ FPKM (number of fragments per kilobase of transcript sequence per million base pairs sequenced) + 1] in a given sample is represented on a blue (low expression) to red (high expression) scale. **(C)** Specifically- and commonly-expressed genes in fertile and sterile flowers.

The numbers of clean reads that mapped preferentially to the assembled unigenes were 110,816,400 in fertile flowers and 113,073,500 in sterile flowers (Table S3). Based on the mapping results, we further estimated the expression levels of these unigenes in terms of FPKM values. We filtered the unigenes with low expression by applying a cut-off of RPKM < 0.3, and the remaining 72,908 and 69,372 unigenes for sterile and fertile flowers, respectively, were deemed to be expressed genes (data not shown). DEGs were determined using DEseq with an adjusted *P* ≤ 0.05. Based on the DEG analysis, 1,908 unigenes were upregulated in sterile flowers, whereas 3,767 unigenes were downregulated in sterile flowers (Table S4; Figures 3A,B). Among those DEGs, 742 DEGs were only expressed in fertile flowers, and only 34 DEGs were specifically expressed in sterile flowers (Table S4; Figure 3C).

**Figure 4:**
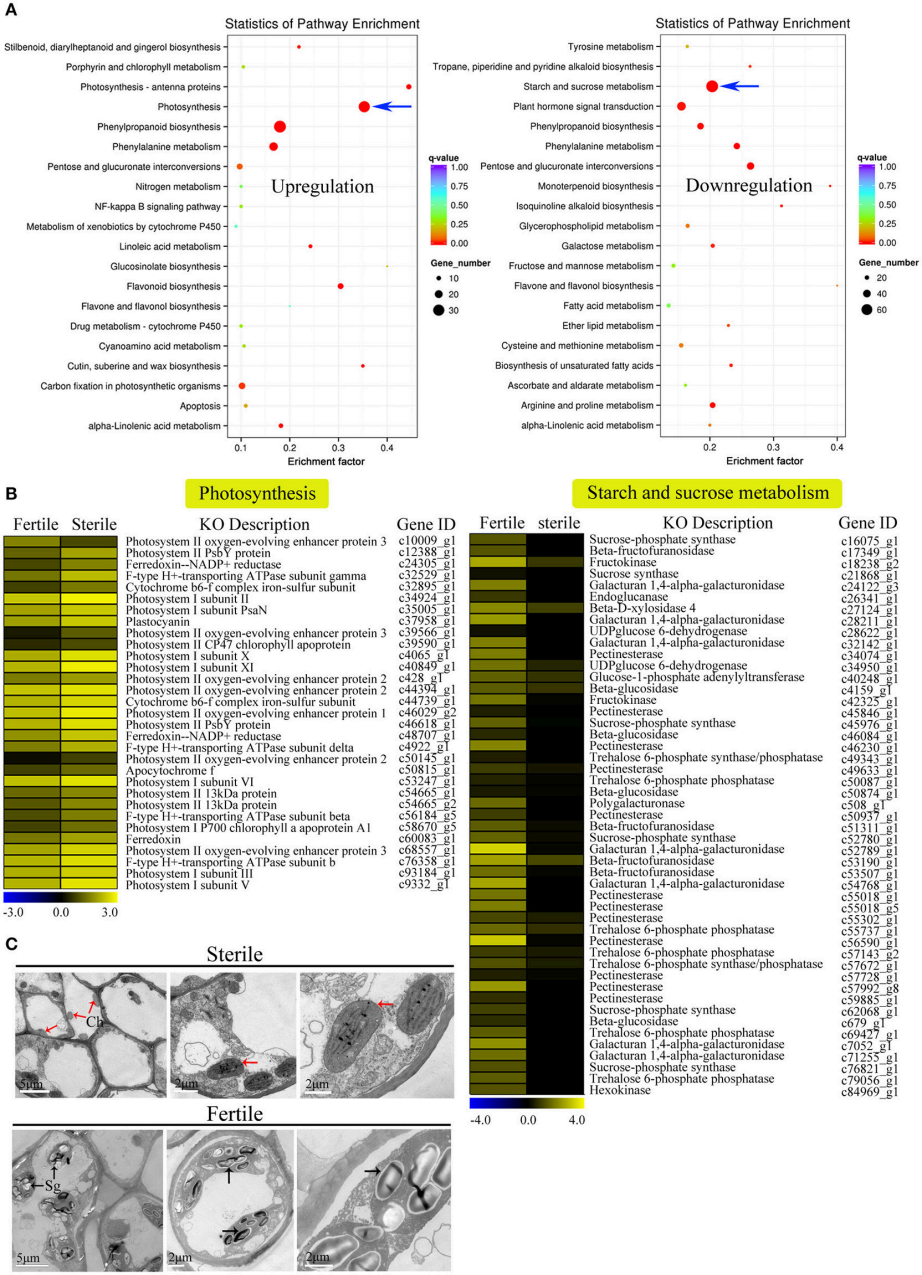
**KEGG enrichment analysis of DEGs (upregulated and downregulated) revealed significantly enriched photosynthesis pathway, starch and sucrose metabolism pathway, and related genes. (A)** Statistics for the top 20 enriched pathways among upregulated and downregulated genes. The degree of KEGG enrichment was determined by the enrichment factor, *q*-value, and gene number. The sizes and colors of spots represent the number of DEGs and the *q*-value. Blue arrow points to the most significantly enriched pathways. **(B)** Expression profile of DEGs involved in photosynthesis (31 DEGs) and starch and sucrose metabolism pathways (49 DEGs) between fertile and sterile flowers. Heatmap shows expression profiles of DEGs. The rows and columns represent genes and samples (fertile and sterile flowers), respectively. Expression differences are shown in different colors. Yellow indicates a high expression level and blue indicates a low expression level. **(C)** Ultrastructural observations of petals of fertile and sterile flowers. Ch, chloroplast; Sg, starch grain. Red and black arrows indicate chloroplasts (sterile flowers) and starch grains (fertile flowers), respectively.

We further performed GO and KEGG enrichment analyses to investigate the biological functions of the DEGs we identified. We found that, in sterile flowers, 1,340 (36.68%) upregulated DEGs were successfully assigned to 45 significantly enriched GO terms, and 2,313 (63.32%) downregulated unigenes were significantly enriched in 42 GO terms (corrected *P* ≤ 0.05; Table S5). Among these significantly enriched GO terms, we focused on some important factors that may be involved in differentiation and development of fertile and sterile flowers. We found that many upregulated DEGs were enriched significantly in photosynthesis (GO: 0015979) and light harvesting (GO: 0009765), and many downregulated genes were enriched significantly in the starch metabolic process (GO: 0005982) and sucrose metabolic process (GO: 0005985) terms (Table 1). Moreover, we found that genes related to pollen development (GO: 0009555) and gametophyte development (GO: 0048229), such as gene homologs of dynamin-related protein 1C (c101321_g1, *DRP1C*), copper transporter 1 (c49933_g1, *COPT1*), spermidine hydroxycinnamoyl transferase (c57651_g1, *SHT*), and transcription factor GAMYB (c57595_g3, *GAM1*) (Table S5), were downregulated significantly in sterile flowers (Table 1). The KEGG enrichment results were similar to the GO-enriched terms and the gene expression profiles. KEGG pathway annotations showed that upregulated and downregulated unigenes were enriched in 131 and 149 KEGG pathways, respectively. We listed the top 20 enriched pathways with the highest representation in a scatter plot (Figure 4A). Of these, photosynthesis was the most significantly enriched pathway among the upregulated DEGs in sterile flowers (corrected *P* ≤ 0.05), and 31 DEGs encoding proteins associated with photosynthesis were identified: for example, ferredoxin-NADP reductase (c48707_g1, *PETH*), photosystem II oxygen-evolving enhancer protein 2 (c428_g1, *PSBP*), and photosystem I subunit XI (c40849_g1, *PSAL*; Figure 4B). The most significantly enriched pathway among downregulated DEGs in sterile flowers was starch and sucrose metabolism. In total, 49 DEGs encoding proteins related to starch and sucrose metabolism, including pectinesterase (c57992_g8, *PME*), beta-glucosidase (c679_g1, *BGLU*), sucrose-phosphate synthase (c45976_g1, *SPS*), and UDP-glucose 6-dehydrogenase (c34950_g1, *UGD*) were identified (Figure 4B). Through ultrastructural observations, we found that chloroplasts were clearly visible in petal cells of sterile flowers, but were only rarely present in fertile flowers. Furthermore, fewer starch grains were contained in the petal cells of sterile flowers than in fertile flowers (Figure 4C). These significant differences in chloroplast and starch grain distributions in petal cells from sterile and fertile flowers confirmed a close correlation between petal organs and the KEGG enrichment analysis and GO-enriched results.

**Table 1:** **GO terms significantly enriched among up- and downregulated DEGs**.

**GO term^a^**	**Description**	**Number in input list**	**Number in BG/Ref^b^**	**Corrected *P***
**UPREGULATION**				
GO:0006464	Cellular protein modification process	196	2,602	3.41E-07
GO:0006118	Electron transport	52	715	0.020327
GO:0015979	Photosynthesis	47	440	0.0001254
GO:0009416	Response to light stimulus	24	145	4.43E-05
GO:0019684	Photosynthesis, light reaction	14	93	0.0086153
GO:0007267	Cell–cell signaling	13	64	0.0016466
GO:0009765	Photosynthesis, light harvesting	8	22	0.00021899
GO:0009638	Phototropism	3	3	0.025256
**DOWNREGULATION**				
GO:0044042	Glucan metabolic process	111	502	2.17E-19
GO:0005984	Disaccharide metabolic process	110	478	2.60E-20
GO:0005985	Sucrose metabolic process	100	442	5.01E-18
GO:0005982	Starch metabolic process	98	441	3.40E-17
GO:0046351	Disaccharide biosynthetic process	14	53	0.0042711
GO:0005992	Trehalose biosynthetic process	14	49	0.0019354
GO:0009555	Pollen development	14	40	0.00066549
GO:0048229	Gametophyte development	14	58	0.030959

a *These significantly enriched GO terms were selected from Table S5 for their important functions in flower development*.

b *BG/Ref, Background/Reference*.

### Identification of candidate DEGs involved in reproductive processes and expression dynamics analysis

**Figure 5:**
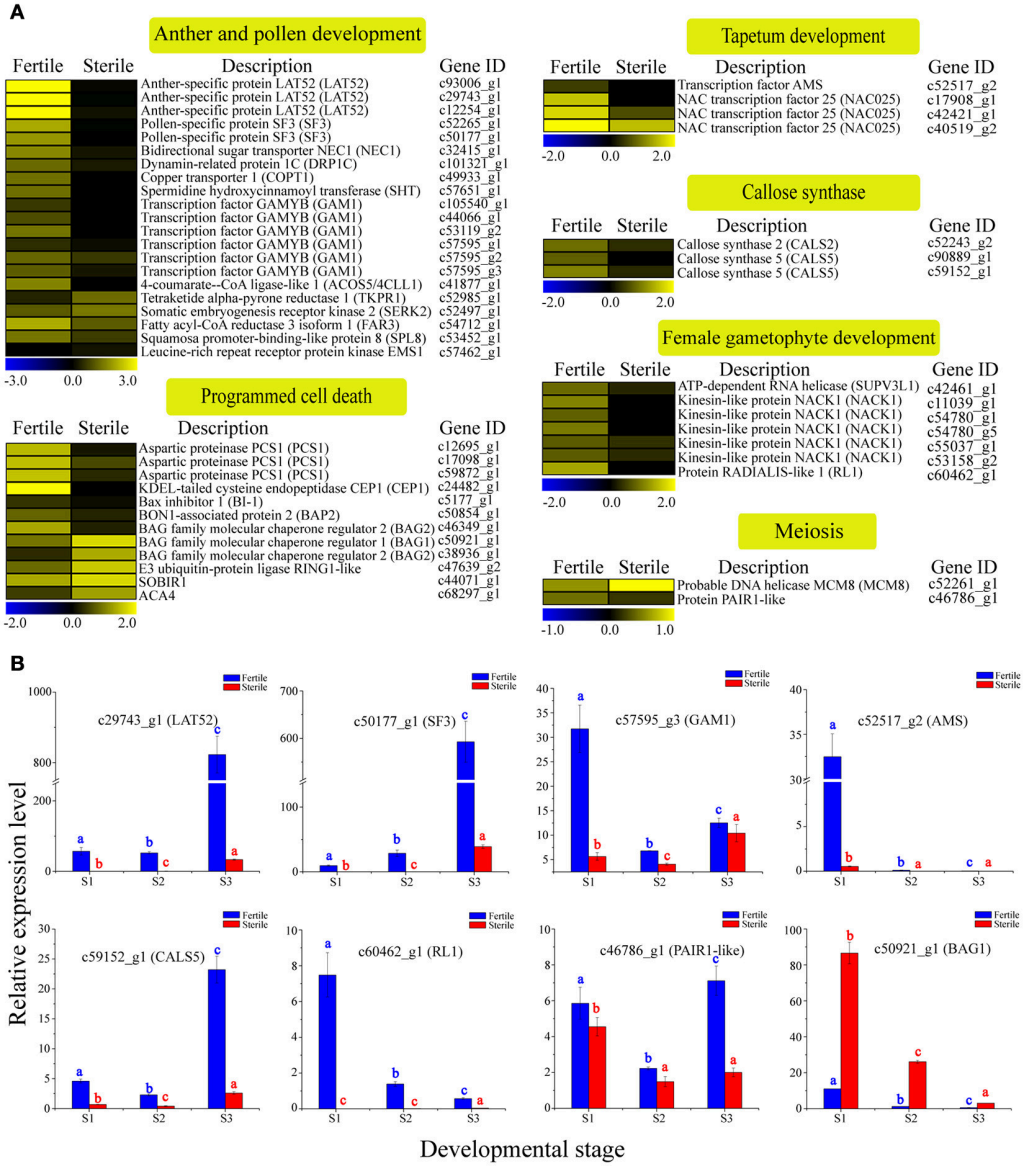
**Expression profile of 49 DEGs involved in reproductive processes between fertile and sterile flowers of *V. macrocephalum* f. *keteleeri* and qRT-PCR analysis. (A)** Heatmap shows expression of genes associated with anther and pollen development, tapetum development, callose synthase, female gametophyte development, meiosis, and programmed cell death (PCD) in RNA-seq samples (fertile and sterile flowers at March 31). The bar represents the scale of the expression levels for each gene in fertile and sterile flowers, as indicated by blue (low expression) and yellow rectangles (high expression). **(B)** qRT-PCR analysis of the expression profiles of eight transcripts during fertile (blue) and sterile flower (red) development. The points (S1–S3) from left to right on the *x*-axis represent different developmental stages. The *y*-axes show relative expression levels analyzed by qRT-PCR. Columns and error bars indicate means and standard deviations of relative expression levels (*n* = 3), respectively. Non-overlapping letters (a–c) indicate significant differences (*P* < 0.05) between fertile (blue letters) or sterile flowers (red letters) from different stages. Significant differences (*P* < 0.05) between fertile and sterile flowers in each stage were also evaluated.

Sterile flowers exhibited degenerated stamens (or anthers) and pistils, which were distinct from fertile flowers with normal stamens and pistils (Figure 1). Thus, we concentrated on genes involved in reproductive processes, including anther and pollen development, tapetum development, callose synthase, female gametophyte development, meiosis, and programmed cell death (PCD), and found that many candidate genes were differentially expressed between sterile and fertile flowers (Table S6; Figure 5A). For example, the homologs of genes involved in another development, such as *LAT52*, which encodes the anther-specific LAT52 protein, showed significantly lower expression in sterile flowers. Most of the homologous regulators related to pollen development, including the bidirectional sugar transporter NEC1 (*NEC1*), myb-like DNA-binding domain transcription factor GAMYB (*GAM1*), pollen-specific protein SF3 (*SF3*), and 4-coumarate-CoA ligase-like 1 (*ACOS5*) were downregulated in sterile flowers. Additionally, gene homologs related to callose synthase, tapetum development, and female gametophyte development, such as callose synthase 2 (*CALS2*), transcription factor ABORTED MICROSPORES (*AMS*), and protein RADIALIS-like 1 (*RL1*), and homologs of meiosis-related PAIR1-like protein (*PAIR1*-like), also showed lower expression in sterile flowers than in fertile flowers. In contrast, the expression levels of all these genes were upregulated significantly in fertile flowers, indicating that these genes are important for the normal development of reproductive organs.

Programmed cell death (PCD) occurs commonly in flowering and reproduction processes, and is required for tapetum and pollen development (Thomas and Franklin-Tong, 2004; Zhang et al., 2014). Thus, we next analyzed the homologs of PCD negative regulators, such as aspartic proteinase PCS1 (*PCS1*); calcium-transporting ATPase 4, plasma membrane-type (*ACA4*); Bax inhibitor 1 (*BI-1*); and tapetal PCD-associated KDEL-tailed cysteine endopeptidase CEP1 (*CEP1*) and found that their expression levels were all low in sterile flowers and higher in fertile flowers. Additionally, BAG family molecular chaperone regulator 1 (*BAG1*) and *BAG2*, which may act as positive regulatory factors in PCD, were upregulated in sterile flowers and downregulated in fertile flowers. These results indicate that these PCD regulators may be associated with the degenerescence of reproductive organs contributing to differentiation and development of sterile and fertile flowers.

To validate the differential expression results, eight DEGs involved in reproductive processes, *LAT52, SF3, GAM1, AMS, CALS5, RL1, PAIR1*-like, and *BAG2*, were selected for qRT-PCR analysis (Figure 5B). The results showed that the relative expression levels of seven key regulators were lower in sterile flowers than in fertile flowers at S2, confirming, in all cases, the differential expression observed with RNA-Seq. Moreover, we compared the changes in these expression levels during the various development stages of sterile and fertile flowers and found that *LAT52, SF3, GAM1, AMS, CALS5, RL1*, and *PAIR1*-like genes showed higher levels of expression from S1 to S3 in fertile than sterile flowers. In contrast, *BAG1* showed a lower level of expression from S1 to S3 in fertile flowers. We further found that the levels of the *LAT52, SF3*, and *CALS5* remained low in S1 and S2 and then increased significantly from S2 to S3 in fertile flowers, whereas *AMS* showed highest expression in S1 and was almost undetectable in S2 and S3, indicating that *LAT52, SF3*, and *CALS5* are involved in maintaining pollen and tapetum development, and *AMS* made a greater contribution to regulating early tapetum development of fertile flowers than of sterile flowers.

### Identification of candidate DEGs associated with cell proliferation and expansion and expression dynamics analyses

**Figure 6:**
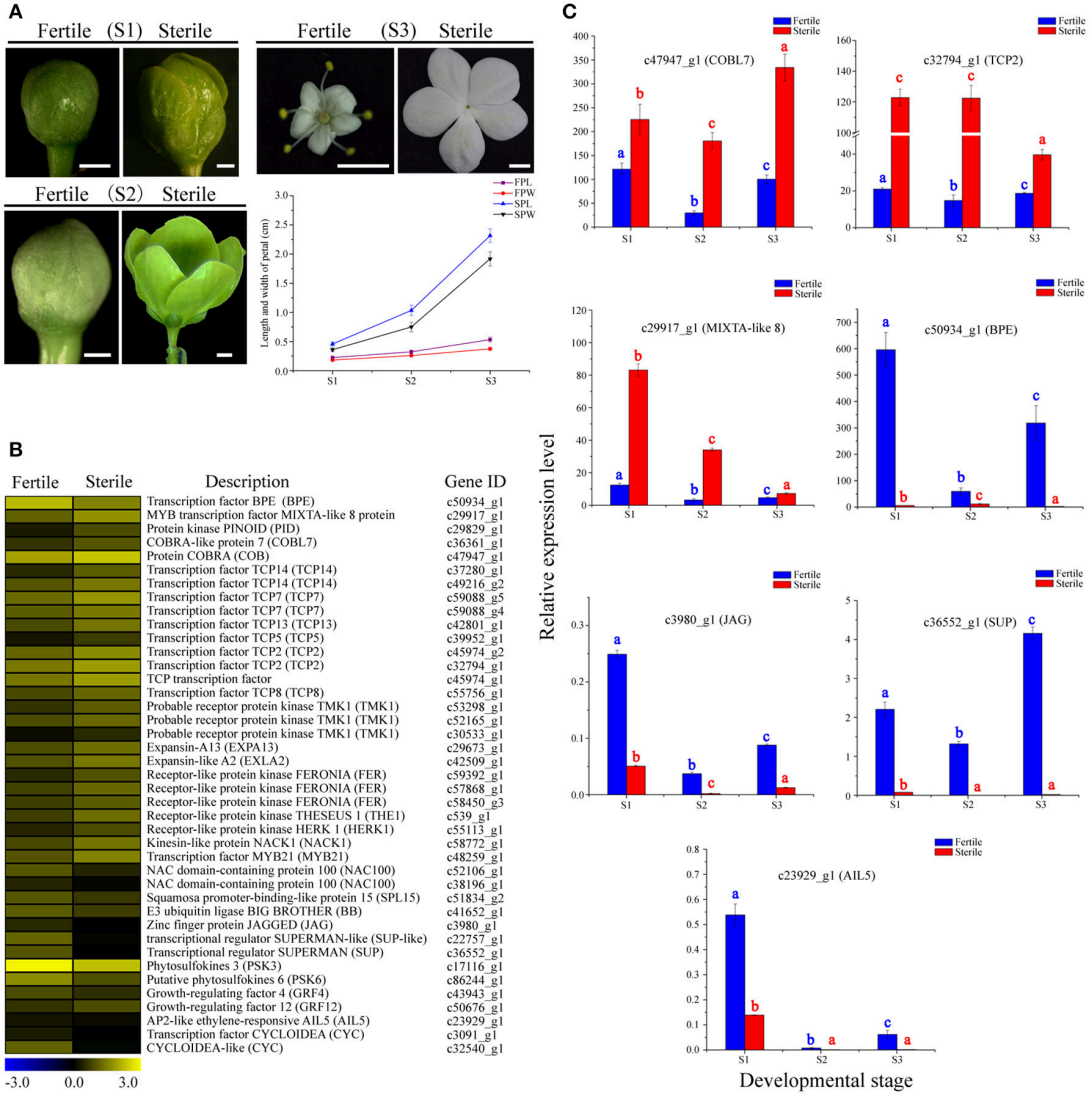
**Expression profiles of 41 DEGs involved in cell proliferation and expansion between fertile and sterile flowers of ***V. macrocephalum*** f. ***keteleeri*** and qRT-PCR analysis. (A)** Morphological analysis of petal development of fertile and sterile flowers. The lengths and widths of petals were measured from stereoscope images of 30 petals at each developmental stage using AutoCAD software. The *x*-axis shows different developmental stages (S1–S3), while the *y*-axes show corresponding measured data from AutoCAD. FPL, length of fertile petal; FPW, width of fertile petal; SPL, length of sterile petal; and SPW, width of sterile petal. Bars = 0.1 cm (S1, S2), 1 cm (S3). **(B)** Heatmap shows expression of genes in RNA-seq samples. The representation of bars is the same as in Figure 5A. **(C)** qRT-PCR analysis of the expression profiles of seven DEGs during fertile (blue) and sterile flower (red) development. The representation of the *x*-axis, *y*-axis, significance tests and error bars are as described in Figure 5B.

Through morphological analyses of petal development at the S1, S2, and S3 stages of fertile and sterile flowers, we found that the lengths and widths of petals in sterile flowers (2.31 ± 0.11 in length; 1.91 ± 0.12 in width at S3) were markedly larger than in fertile flowers (0.53 ± 0.04 in length; 0.37 ± 0.02 in width at S3; Figure 6A). Given the rapid expansion in sterile flower petals, compared with fertile flowers, we investigated genes associated with cell proliferation and expansion. In total, 41 candidate DEGs were identified and most of them were upregulated in sterile flowers, such as genes encoding expansin-like A2 (*EXPA2*), *EXPA13*, protein COBRA (*COB*), receptor protein kinase TMK1 (*TMK1*), receptor-like protein kinase FERONIA (*FER*), THESEUS 1 (*THE1*), kinesin-like protein NACK1 (*NACK1*), and MIXTA-like 8 protein (Table S7; Figure 6B). Additionally, a negative regulator of cell proliferation and expansion, BIG PETAL (*BPE*, Varaud et al., 2011), was identified and showed lower expression levels in sterile flowers. In particular, we detected many genes of the TCP family, which plays significant roles in the morphological characteristics of the floral organ (Yang et al., 2015), and found their expression levels were also higher in sterile flowers than in fertile flowers. These included *TCP2, TCP5, TCP7, TCP8, TCP13, TCP14*, and *TCP15*. These results suggested that these TCP family members may contribute to controlling floral morphology in *V. macrocephalum* f. *keteleeri*. However, we also detected other candidate genes associated with proliferation and expansion, including the transcriptional regulators SUPERMAN (*SUP*), AINTEGUMENTA-like 5 (*AIL5*), zinc finger protein JAGGED (*JAG*), and squamosa promoter-binding-like protein 15 (*SPL15*), and found that they were upregulated in fertile flowers. We propose that these upregulated genes play important roles in controlling reproductive organ development.

We examined the expression level changes of seven key cell proliferation- and expansion-related DEGs, *BPE, COBL7, TCP2, MIXTA*-like 8, *JAG, AIL5*, and *SUP*, by qRT-PCR during the development stages of sterile and fertile flowers (Figure 6C). Our results showed that the expression levels of *COBL7, TCP2*, and *MIXTA*-like 8 genes were higher during the development stages of sterile flowers vs. fertile flowers. In contrast, significantly lower expression levels of *BPE, AIL5, JAG*, and *SUP* were observed in sterile flowers. We found that the expression levels for the regulatory factor *TCP2* and *MIXTA*-like 8 were highest in sterile flowers at S1 and S2, and then decreased markedly in S3, consistent with the rapid development of sterile flowers from S1 to S2. In contrast, the level of *BPE*, acting as a negative regulatory factor, showed highest expression levels at S1 in fertile flowers, and then declined significantly in S2 and S3, indicating its important role in regulating floral organ development. Our qRT-PCR results were also generally consistent with the RNA-Seq data, despite some differences in expression levels.

### Identification of candidate DEGs related to phytohormone signaling and expression dynamics analyses

**Figure 7:**
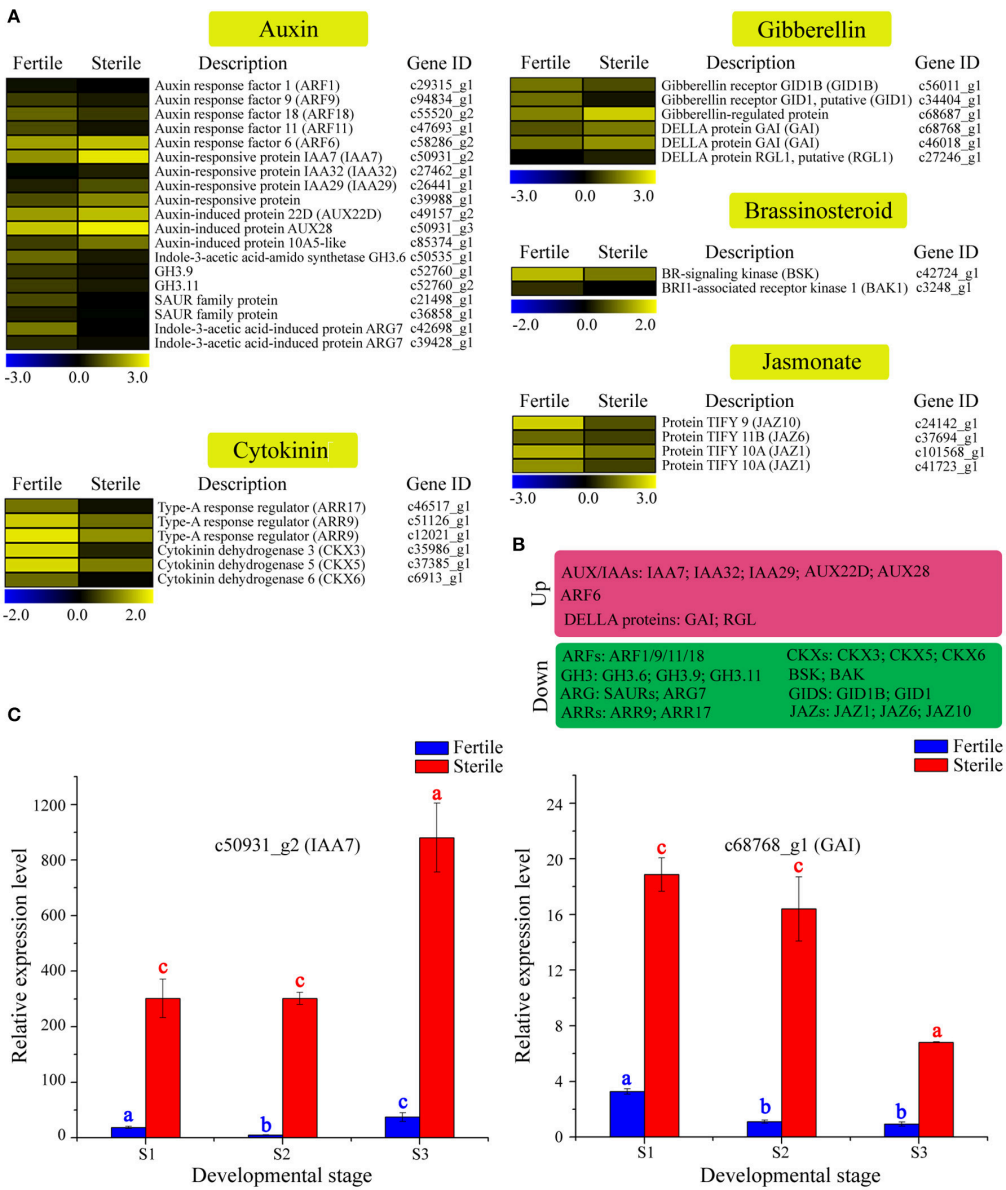
**Expression profile of 37 DEGs involved in phytohormone signaling between fertile and sterile flowers of ***V. macrocephalum*** f. ***keteleeri*** and qRT-PCR analysis. (A)** Heatmap shows expression of genes associated with auxin, cytokinin, brassinosteroid, gibberellin, and jasmonate signaling in RNA-seq samples. The representation of the bars is as described in Figure 5A. **(B)** Overview of DEGs, summarized by upregulation and downregulation in sterile flowers. **(C)** qRT-PCR analysis of two transcripts during fertile (blue) and sterile flower (red) development. The representation of the *x*-axis, *y*-axis, significance tests and error bars are as described in Figure 5B.

Phytohormone signaling plays a vital role in regulating floral organ growth and reproductive processes (Song et al., 2013). We identified many homologous genes involved in phytohormone signaling, including genes related to auxin, cytokinin, brassinosteroid, gibberellin, and jasmonate that showed differential expression between sterile and fertile flowers (Table S8; Figures 7A,B). For example, in the auxin signaling pathway, most of the genes encoding auxin-response factors (ARFs), the indole-3-acetic acid-amido synthetase GH3 family, and SAUR family proteins were upregulated in fertile flowers. In contrast, the auxin-responsive proteins (AUX/IAA), involved in auxin signaling function as repressors of early auxin response genes, were downregulated in fertile flowers. In the gibberellin and jasmonate signaling pathways, genes encoding the protein TIFY (*JAZ1/6/10*) and the gibberellin receptor GID1 (*GID1*) were upregulated in fertile flowers, whereas the DELLA proteins (*GAI, RGL1*), which act as repressors of the gibberellin signaling pathway (Cheng et al., 2004), were downregulated. Additionally, the type-A response regulator genes (*ARR9, ARR17*), as negative regulators in the cytokinin signaling pathway, had higher expression levels in fertile flowers. These results indicate that auxin, gibberellin, jasmonate, and cytokinin signaling-related genes are involved in maintaining fertility/infertility or promoting the developmental divergence between fertile and sterile flowers.

We further selected two key DEGs (*IAA7* and *GAI1*) involved in auxin and jasmonate signaling to assess their expression level changes during different development stages (Figure 7C). qRT-PCR analysis showed that the expression of *IAA7* decreased slightly from S1 to S2 in sterile flowers (not significant), and was highest in S3. In contrast with *IAA7*, in sterile flowers, *GAI* expression was highest in S1 and declined from S1 to S3, indicating the genes' involvement in auxin and gibberellin signaling in sterile flower development.

### Analysis of putative TFs and other regulators involved in flower development and expression dynamics analyses

**Figure 8:**
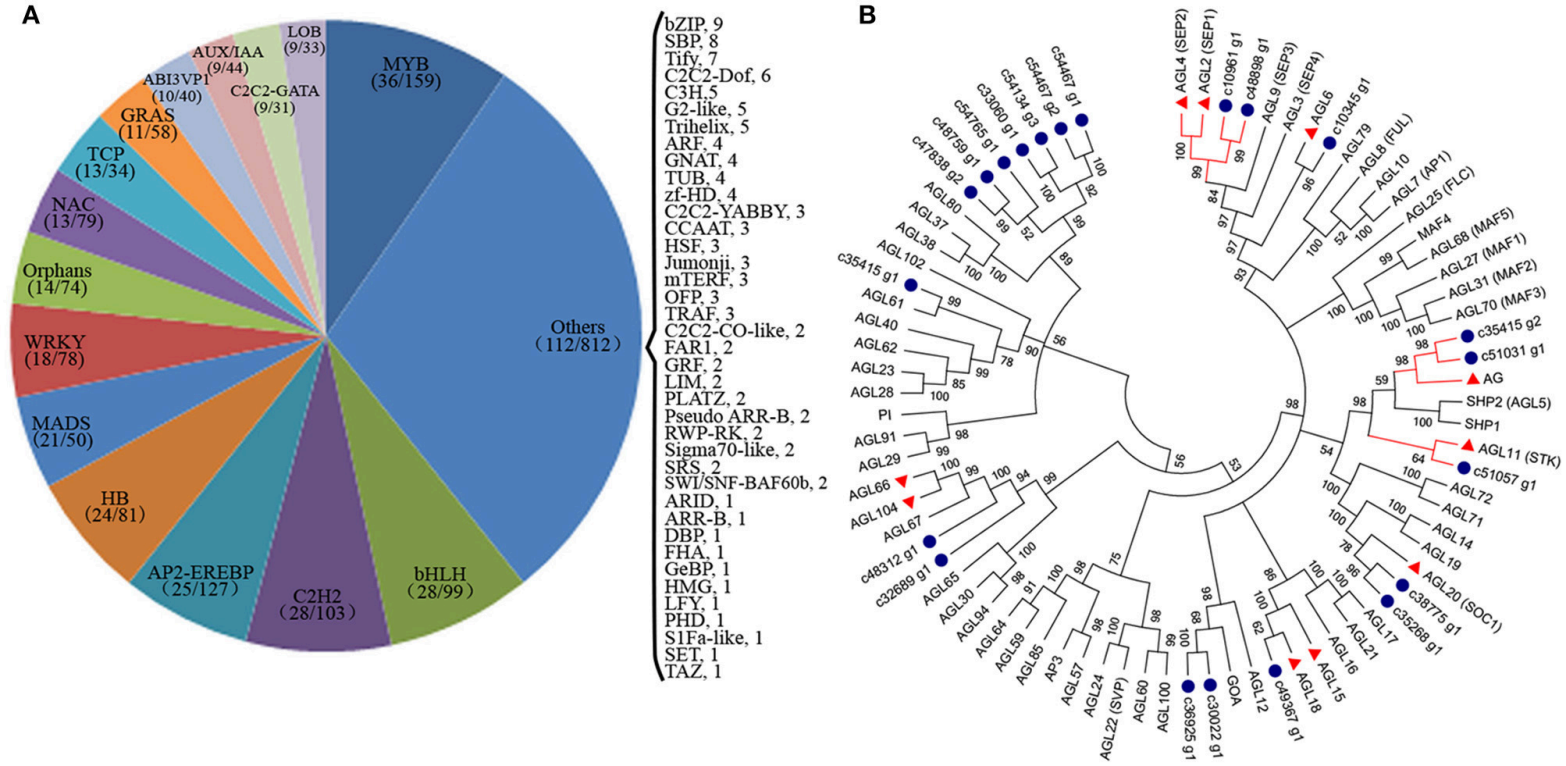
**Analysis of transcription factors (TFs) during flower development in ***V. macrocephalum*** f. ***keteleeri***. (A)** Distribution of TF families. TFs were identified in fertile and sterile flowers of *V. macrocephalum* f. *keteleeri*. Numbers in the parentheses after each TF family indicate the number of differentially expressed TFs (first number) and all members of this TF family identified by RNA-Seq (second number). **(B)** Phylogenetic analyses of MADS-box genes. A phylogenetic tree of 21 MADS-box genes from *V. macrocephalum* f. *keteleeri* and 60 MADS-box genes from *A. thaliana*. Blue circles indicate genes from *V. macrocephalum* f. *keteleeri*. The red clades show C, D, and E class MADS-box genes associated with floral organ identify. Red triangles correspond to floral regulators in *A. thaliana*.

TFs are key regulatory proteins that play important roles in regulating gene expression in various plant biological processes, such as flower development, secondary metabolism, and responses to abiotic and biotic stresses (Riechmann and Ratcliffe, 2000; Singh et al., 2002; Yang et al., 2012). We found that 2,072 genes were putatively identified as TFs and associated with 79 TF families in the integrative plant transcription factor database (PlnTFDB; Pérez-Rodríguez et al., 2009). Of them, the most abundant TF family was the MYB superfamily (159, 7.68%), followed by AP2-EREBP (127, 6.13%), C2H2 (102, 4.93%), and bHLH (99, 4.78%; Figure 8A). In total, 50 TFs were associated with the MADS-box family, which are regarded as flower development regulators (Table S9). For example, gene homologs encoding DEFICIENS (*DEF*) and GLOBOSA (*GLO*) proteins were identified as B class genes, and *AGAMOUS* (*AG*) homologs have been identified as C class genes in *V. macrocephalum* f. *keteleeri*. To obtain a more comprehensive class of ABCDE gene homologs in this species, we selected 21 MADS-box genes and the 60 MADS-box genes of *A. thaliana* to perform a phylogenetic analysis (Figure 8B). This analysis showed that two orthologs (c48898_g1, c10961_g1) of *SEPALLATA 1* (*SEP1*) and *SEP2* formed a well-supported clade as class E gene homologs. The c35415_g2 and c51031_g1 transcripts were identified as *AGAMOUS* (*AG*) family genes, defined as class C gene homologs. Other MADS-box TFs of *V. macrocephalum* f. *keteleeri* also appeared to cluster with strong support with particular genes from *A. thaliana*, such as *SOC1, MADS6*, and *AGL18*-like genes. On the basis of this orthology analysis, we constructed putative orthologs of ABCDE-class genes, to characterize floral organ development in *V. macrocephalum* f. *keteleeri* (Table 2).

**Table 2:** *****V. macrocephalum*** f. ***keteleeri*** unigenes that show homology with ABCDE-class genes**.

**Category**	**Transcript ID**	**Orthologous gene**
A class	c15401_g1	Floral homeotic protein APETALA 1 (*AP1*)
	c79121_g1	
	c56198_g1	
	c31141_g1	Floral homeotic protein APETALA 2 (*AP2*)
	c44268_g1	
	c48961_g2	
	c50326_g1	
	c56440_g2	
B class	c44362_g2	Floral homeotic protein PISTILLATA (*PI*)
	c101101_g1	Floral homeotic protein GLOBOSA (*GLO*)
	c76268_g1	Floral homeotic protein DEFICIENS (*DEFA*)
	c47136_g1	
C class	c35415_g2	Floral homeotic protein AGAMOUS (*AG2*/*AG*)
	c51031_g1	
	c35415_g1	
D class	c51057_g1	Agamous-like MADS-box protein AGL11 (*STK*)
E class	c10961_g1	Developmental protein SEPALLATA 1/2 (*SEP1*/*2*)
	c48898_g1	

**Figure 9:**
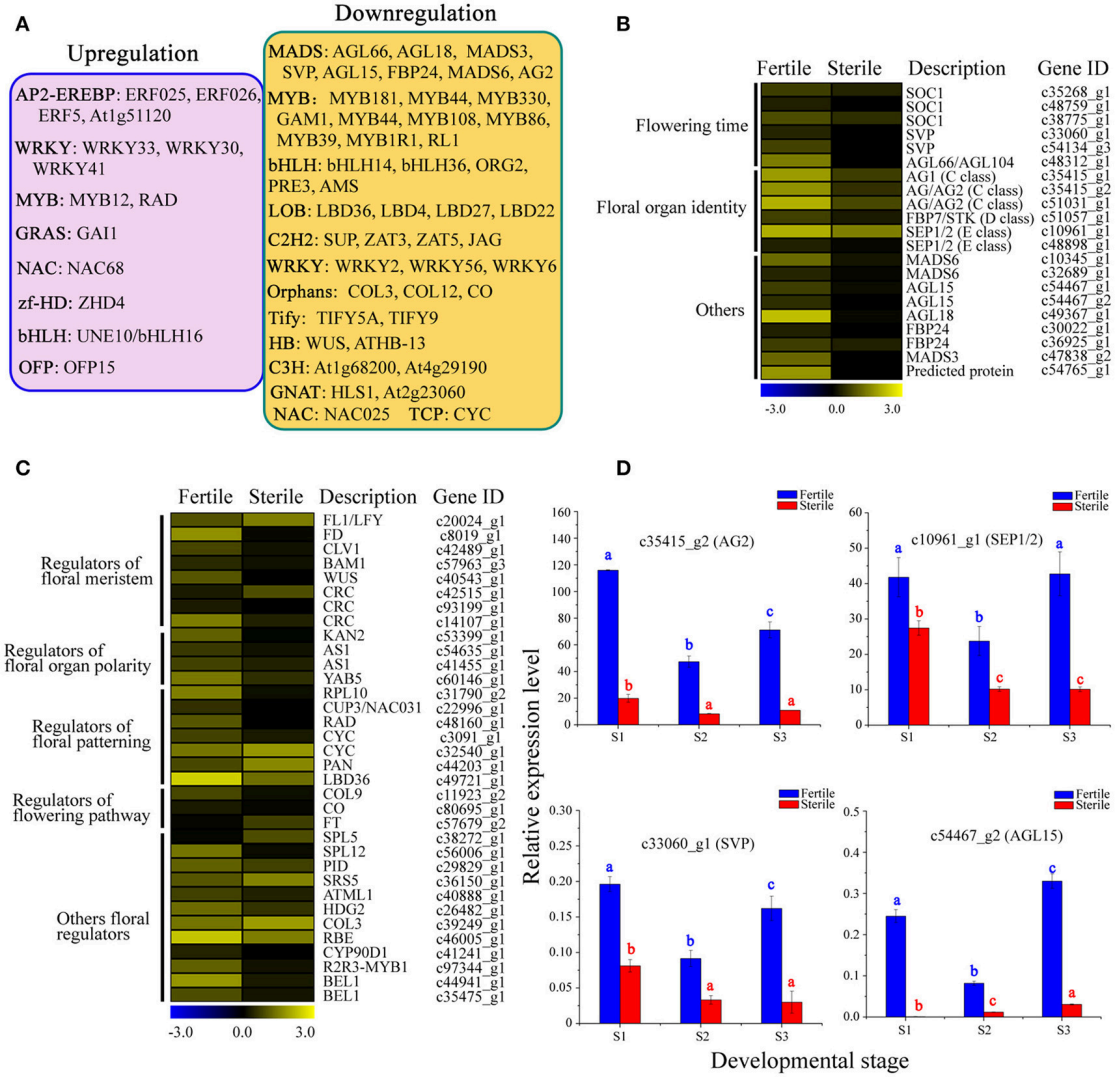
**Analysis of differentially expressed TFs and other floral regulators between fertile and sterile flowers. (A)** Representative functions and genes showing different transcription factor families for fertile and sterile flowers. Significantly upregulated (pink) and downregulated (orange) TFs in sterile flowers were screened using a cut-off of |log2(ratio)| ≥ 4. **(B)** Expression profile of MADS-box TFs involved in flowering time, floral organ identity, and other roles. The representation of the bar is as described in Figure 5A. **(C)** Expression profile of other differentially expressed TFs and other genes involved in floral meristem, floral patterning, floral organ polarity, floral patterning, flowering pathway, and other processes. The representation of the bar is as described in Figure 5A. **(D)** qRT-PCR analysis of four transcripts during fertile (blue) and sterile flower (red) development. The representation of the *x*-axis, *y*-axis, significance tests and error bars are as described in Figure 5B.

We next performed a differential expression analysis of the identified TFs, and found that 377 TFs could be classified into 54 TF families displaying differential expression between fertile and sterile flowers. The largest numbers of differentially expressed TFs were in the MYB family (36), followed by bHLH (28), C2H2 (28), and AP2-EREBP (25). We performed a screen on differentially expressed TFs (|log2(ratio)| ≥ 4) to identify those that were significantly upregulated or downregulated in sterile flowers (Figure 9A). We found that among the differentially expressed TFs, the majority were significantly downregulated in sterile flowers, while the majority, including MYB and MADS family members, were upregulated in fertile flowers (Figure 9A). All the differentially expressed MADS family TFs showed significantly lower expression in sterile flowers and high expression in fertile flowers (Figure 9B). We summarized all differentially expressed MADS-box genes that may be involved in controlling flowering time, floral organ identify, and other functions in Figure 9B. We also identified many other regulators involved in floral meristem, floral patterning, floral organ polarity, floral patterning, flowering pathway, and others showing differential expression profiles between fertile and sterile flowers (Table S10; Figure 9C). Most of these TFs and genes also had lower expression levels in sterile flowers and higher levels in fertile flowers.

Furthermore, we performed qRT-PCR experiments to determine the expression patterns of key regulators, including *AG1, SEP1*/*2, SVP*, and *AGL15*, which are involved in regulating flowering time, floral organ development, and floral meristem during the different developmental stages (Figure 9D). We found that the expression levels of *AG1* and *SEP1*/*2* of classes B and E, respectively, declined from S1 to S3 in sterile flowers. Additionally, the levels of the negative regulatory factor genes *SVP* and *AGL15*, which are associated with flowering time, declined from S1 to S2 in fertile flowers and then increased in S3. However, all these key genes showed lower expression levels in sterile flowers than in fertile flowers, indicating their involvement in floral organ identify and flowering time.

## Discussion

### *De novo* assembly and transcriptome annotation

*De novo* transcriptome analyses have been used widely for flowering plants without a reference genome to discover genes and their expression patterns involved in flower and reproductive developmental processes. Previous studies with floral transcriptomes from different stages and tissues have contributed to identifying new floral-expressed genes (Zhang et al., 2014), floral biomarker genes, stage-specific genes, tissues-specific genes (Vining et al., 2015), transcription factors, lineage-specific genes (Bhide et al., 2014; Zhang et al., 2014), and flowering time regulators (Fan et al., 2015). Sterile and fertile flower differentiation and formation are driven by adaptive and selective stress, and as an important evo-devo phenotype associated with flower shape, size, and fertility. For example, from transcriptome profile analyses of fertile and sterile floral buds from plants with cytoplasmic male sterility (CMS) or genic male sterility (GMS) such as *Brassica napus* (An et al., 2014), cotton (Yang et al., 2014), and *Capsicum annuum* (Chen et al., 2015), changes in expression patterns of some genes involved in anther and pollen development have been identified between fertile and sterile floral buds. However, a comparative global expression analysis of fertile vs. sterile flowers has been lacking.

Because the inflorescence of *V. macrocephalum* f. *keteleeri* contains distinct sterile and fertile flowers, comparisons of sterile and fertile flower materials within one inflorescence enable analyses in a consistent genetic background. With no available genomic information for this species, we used RNA-seq to obtain large numbers of paired-end clean reads (34.4 G) from sterile and fertile flowers, and constructed more comprehensive transcripts (105,683 unigenes). This large number of reads produced more unigenes than those generated from some perennial shrubs (Gao et al., 2015; Zheng et al., 2015) and increased the coverage depth of the transcriptome, improving the *de novo* assembly and sequencing accuracy.

Further annotation of the unigenes revealed that reproductive processes, cell growth and death, and development-associated genes participated in the differentiation and development of fertile and sterile flowers. However, over 55% of unigenes still had no hits in public databases, which may be attributable to many short sequences; moreover, these unmatched unigenes might represent genes specific to *V. macrocephalum* f. *keteleeri*.

### Global changes in gene expression reveal significantly enriched pathways in fertile and sterile flowers

Floral organ differentiation and development are highly regulated through temporal and spatial gene expression, with each organ having distinct transcriptomes (Zhang et al., 2014). Because the fertile flowers of *V. macrocephalum* f. *keteleeri* possess normal pistils and stamens, vs. the sterile flowers with abnormal reproductive components, thus we considered that some DEGs between fertile and sterile flowers would be associated with reproductive processes and/or plant fertility. Although over 5,000 DEGs between fertile and sterile flower development were found in this study, the key or upstream regulators triggering divergence may not be included in S2, as such regulators are likely expressed in an earlier stage. Therefore, the DEGs identified may refer to the downstream genes or regulators that maintain reproductive units underlying differentiation and development of fertile and sterile flowers during the rapid development stage.

Previous studies have confirmed that some genes, such as *COPT1*, sucrose-phosphate synthase (*SPS*), *SHT, GAM1*, and sucrose synthase (*SUS*), are required for pollen development and starch and sucrose metabolism (Sancenón et al., 2004; Park et al., 2008; Lin et al., 2014). In the present study, these genes were significantly downregulated in sterile flowers compared to fertile flowers, suggesting that they may participate in the degeneration and stagnation of stamens in sterile flowers during their differentiation and development.

Regarding upregulated DEGs in sterile flowers, many photosynthesis-related genes, such as *PSBP* and *PETH*, function in electron transfer in photosynthesis activity (Ishihara et al., 2007; Lintala et al., 2007). This result was consistent with the ultrastructural observations that more chloroplasts were distributed in the petal cells of sterile flowers, indicating that the sterile flowers had relatively high photosynthesis capabilities. Considering that sterile flowers have much greater size and higher biomass than do fertile flowers, we speculate that the process of flower formation and petal expansion in sterile flowers may be partially attributed to their higher photosynthetic capabilities. After all, having more photosynthetic products is beneficial to supplying the energy and materials needed to construct larger floral organs in sterile flowers.

### Expression level changes in genes involved in reproductive processes in fertile and sterile flowers

Sexual reproduction requires a developmental phase transition, and results in the formation of flowers with highly specialized organs, including anther-bearing stamens and ovule-bearing carpels (Xing et al., 2010). Within these organs, cells are recruited to undergo meiotic divisions to form male and female gametophytes. Many of the genes and regulatory pathways controlling anther and pollen development, meiosis, and female gametophyte development have been characterized (Irish, 2010). Molecular and genetic studies have found that the altered function of some genes can result in severe reductions in fertility. For example, knocking out the *CALS5* gene, encoding a callose synthase that is essential for exine formation in the pollen wall, can reduce *Arabidopsis* fertility (Dong et al., 2005). Similarly, mutations in *Arabidopsis EXTRA SPOROGENOUS CELLS/EXCESS MICROSPOROCYTES1* (*EMS1*/*EXS*) can cause abnormal tapetum development and result in male sterility (Canales et al., 2002). Ectopic expression or altered function of some other genes, including *PCS1, PAIR1, PAIR2, PAIR3*, and *MCM8*, also can lead to a failure in anther dehiscence and fertility, as well as meiosis (Ge et al., 2005; Nonomura et al., 2006; Yuan et al., 2009; Crismani et al., 2013), suggesting their important roles in reproductive organ development. Recently, many genes responsible for anther and pollen development in the CMS and GMS systems of *B. napus* and *Citrullus lanatus* have been identified and they showed differential expression levels between fertile and sterile flower buds (An et al., 2014; Rhee et al., 2015).

Here, our RNA-seq and qRT-PCR results revealed that many homologs of genes involved in anther and pollen development were significantly downregulated in sterile flowers, including *CALS5, AMS, GAM1, LAT52 SF3, NEC1, ASCO5, SHT*, and *DRP1C*, suggesting that they are potential factors causing stamen degradation in sterile flowers. Similar expression changes were also observed in female gametophyte development-associated genes, such as *NACK1*, ATP-dependent RNA helicase (*SUV3*), and *RL1*, which likely give rise to abnormal female gametophytes, subsequently resulting in the collapse of pistils in sterile flowers.

In particular, qRT-PCR results indicated that PCD-related *BAG1* was significantly expressed at higher levels from S1 to S3 in sterile flowers, suggesting that the gene may cause rapid PCD in degenerated stamens and pistils of sterile flowers. These results also suggest that some genes have conserved roles in regulating floral organ formation, and these reproduction-associated genes are involved in regulating fertility and sterility differentiation through temporal and spatial gene expression patterns.

### Expression level changes in genes involved in cell proliferation and cell expansion in fertile and sterile flowers

Flowers exhibit various colors, shapes, and sizes, in which petal or flower size is an important attractive characteristic for pollinators. The final size of a flower or an organ depends largely on cell proliferation and cell expansion (Powell and Lenhard, 2012; Czesnick and Lenhard, 2015). Some genes related to cell proliferation and expansion have been identified (Powell and Lenhard, 2012; Czesnick and Lenhard, 2015). For example, a petal-specific transcription factor, *BIGPETAL* (*BPEp*), can interfere with petal cell expansion by interaction with *AUXIN RESPONSE FACTOR8* (*ARF8*) (Varaud et al., 2011). Similarly, the *MIXTA*-like genes have been demonstrated to regulate petal epidermal conical cell differentiation in multiple plant species (Perez-Rodriguez et al., 2005; Weng et al., 2011). In particular, a cascade of transcription factor Class II TCPs and GRFs (growth-response factors) is involved in promoting cell proliferation, and class I TCPs, including *TCP14, TCP15*, and *TCP20*, also modulate cell proliferation and expansion in an organ, suggesting essential roles in controlling organ growth and size (Czesnick and Lenhard, 2015).

In this study, homologs of genes associated with cell proliferation and expansion, such as *COBL, MIXTA*-like 8, *TMK1, EXPA2, EXPA13*, and TCP family members, were upregulated in sterile flowers, indicating that these genes may be associated with the petal size of sterile flowers. Further qRT-PCR analyses revealed that *TCP2* and *MIXTA*-like 8 were significantly highly expressed in S1 and S2 in sterile flowers, suggesting that they may be responsible for regulating cell proliferation or differentiation in rapid petal expansion. In contrast, a homolog of the negative regulator *BPE* showed significantly lower expression from S1 to S3 in sterile flowers, and much lower expression in S2 in sterile flowers, which is consistent with rapid petal expansion and suggests its key role in forming larger petals in sterile than in fertile flowers.

### Expression level changes in genes involved in phytohormone signaling in fertile and sterile flowers

Phytohormones including auxins, gibberellins (GA), cytokinins, brassinosteroids, abscisic acid, and jasmonates all play important roles in the regulation of flower or reproductive development (Yuan and Zhang, 2015). For example, most of the mutants in jasmonate biosynthesis are male sterile and can be rescued by the application of JA (Song et al., 2013; Yuan and Zhang, 2015). Similarly, brassinosteroid can regulate key genes in anther and pollen development (Ye et al., 2010). Increasing evidence indicates that the coordinated actions of jasmonate, auxin, gibberellin, cytokinin, and brassinosteroid play essential roles in the regulation of stamen development in *Arabidopsis* (Song et al., 2013).

In this study, our RNA-seq data revealed that many homologs of genes or proteins involved in auxin (e.g., ARFs, *GH3*, and SAUR family proteins), jasmonate (e.g., JAZs) and gibberellin (e.g., *GID1* and *GIDB*) signaling were downregulated in sterile flowers, in coordination with the downregulation of several genes related to fertility, implying that they may be involved in regulating fertility and sterility differentiation of *V. macrocephalum* f. *keteleeri* flowers. Similar expression patterns for cytokinin signaling-related negative regulators were also seen in sterile flowers, which is consistent with the upregulation of several genes related to cell proliferation and expansion. We propose that cytokinin-associated genes may be a factor in petal expansion through cell proliferation and expansion, contributing to flower size.

### Transcriptional regulation in fertile and sterile flower development

TFs are a group of proteins that act by activating or repressing the expression of downstream target genes; they play important roles in regulating flower development (Qu and Zhu, 2006). Previous studies in the model species *A. thaliana* and *Antirrhinum majus* have identified many TFs from various TF families involved in flower development, such as MADS, bHLH, and MYB family members (Egea-Cortines et al., 1999; Riechmann and Ratcliffe, 2000; Jin et al., 2015). Most *Arabidopsis* MADS family TFs were detected predominantly in flowers (Egea-Cortines et al., 1999; Ó'Maoiléidigh et al., 2014). They are also major components in the classical ABCDE model, and specific combinations of ABCDE genes correspond to the identity of each concentric whorl of sepals (A+E), petals (A+B+E), stamens (B+C+E), carpels (C+E), and ovules (D+E) (Pelaz et al., 2000; Theissen and Melzer, 2007). BHLH family proteins are one of the largest families of TFs, and many of them have been characterized functionally in plants (Carretero-Paulet et al., 2010). The gene *SPATULA*, encoding a bHLH TF, has been shown to be involved in controlling floral-organ formation as well as the morphogenesis of sepals, petals, and stamens in *Arabidopsis* and rice (Li et al., 2006; Groszmann et al., 2010).

In sterile and fertile flowers, most MYB, bHLH, AP2-EREBP, C2H2, and MADS family TFs were highly expressed, suggesting essential roles in regulating *V. macrocephalum* f. *keteleeri* flower development. Further phylogenetic analyses of MADS-box family genes revealed some important floral regulators including ABCDE-class homologous genes, which may contribute to floral organ identification and further functional research in the floral differentiation and development of the genus *Viburnum*. Moreover, RNA-seq and qRT-PCR results indicated many differentially expressed TFs, including MADS-box family members, and showed uniform lower expression in sterile flowers, suggesting probable functions in fertility degeneration in sterile flowers.

## Conclusion

We constructed a transcriptome library from *V. macrocephalum* f. *keteleeri* and obtained large sets of transcript data from its flowers. We found that genes that were differentially expressed between fertile and sterile flowers were involved primarily in photosynthesis, starch and sucrose metabolism, pollen development, female gametophyte development, phytohormone signaling, and cell proliferation and expansion. Additionally, many transcription factors, including MADS-box genes, were involved in fertile vs. sterile flower differentiation. Our results showed involvement of comprehensive transcriptional regulation networks related to flower fertility and size in regulating differentiation and development of fertile and sterile flowers in *V. macrocephalum* f. *keteleeri*.

## Author contributions

ZL and BJ carried out the design of the study and drafted the manuscript. ZL and JX performed the experimental work and data analysis. JX, LZ, JC, and QH participated in sample collection, RNA extraction, quantitative RT-PCR, and data analysis. ZL, LW, BJ, and WL revised the manuscript. All authors read and approved the final manuscript.

## Funding

This work was financially supported by the Three New Forestry Engineering Foundation of Jiangsu Province (No. LYSX(2011)41, LYSX[2016]55), Natural Science Foundation of Jiangsu Province (No. BK20131228).

### Conflict of interest statement

The authors declare that the research was conducted in the absence of any commercial or financial relationships that could be construed as a potential conflict of interest.
