# Supplementary material for: Transcriptomic Analysis Reveals Mechanisms of Sterile and Fertile Flower Differentiation and Development in Viburnum macrocephalum f. keteleeri
Source: Front Plant Sci. 2017 Mar 1;8:261. doi: 10.3389/fpls.2017.00261 (PMC5331048; doi:10.3389/fpls.2017.00261)
Supplement: Supplementary file 1 [file Table1.DOC]

**Table S1.** Primer sequences for qRT-PCR.

| > c29743_g1 Anther-specific protein LAT52 (LAT52)  F: CACACCAAGAACCGCTACG  R: GCACACACATACAAGAGAAGTGG |
| --- |
| > c50177_g1 Pollen-specific protein SF3 (SF3)  F: TCCTATGCTGCTCTGGATGG  R: CATTGCTTCTTTCGGTGGAC |
| > c57595_g3 Transcription factor GAMYB (GAM1)  F: TGAGCCGCATAACAAATGAG  R: CAAAACAACACCTCCACTCG |
| > c52517_g2 Transcription factor ABORTED MICROSPORES (AMS)  F: CGATGGGTTTCGTGTTAGC  R: TGAAGTTGATGGAGGCTTTG |
| > c59152_g1 Callose synthase 5 (CALS5)  F: GACTCCCAACTCTTGCTTGC  R: TGTTTGCTCCTTTCCTCTTCA |
| > c60462_g1 Protein RADIALIS-like 1 (RL1)  F: TTTTTAGCAGCAGTTGGGGTA  R: ATTGGTGAGCGATGTGAATG |
| > c46786_g1 Protein PAIR1-like  F: CATTTTCTCTTTCGCCTCCT  R: TGAAGGTTTTTCTTGCTTGC |
| > c50921_g1 BAG family molecular chaperone regulator 1 (BAG1)  F: GCAAATCCAAACCCAAGAAG  R: AGCAGCAGGAGCAGATGAAT |
| > c47947_g1 COBRA-like protein 7 (COBL7)  F: GCAGACAACGGACGCTT  R: TGTGCCGCAAAGGAAGT |
| > c32794_g1 Transcription factor TCP2 (TCP2)  F: GAAAGCGGTATGGAGATGGA  R: GTCGTCGGGGTATTGTTCA |
| > c29917_g1 MYB transcription factor MIXTA-like 8 protein  F: CTCAAATGTAATCCACCACCAG  R: TCAGAGTAACACAGGGAAGACG |
| > c36552_g1 Transcriptional regulator SUPERMAN (SUP)  F: GGGTTAGGGTTAGGGTTAGGA  R: ACAGGAGAGATAGAGCGAGGA |
| > c41652_g1 E3 ubiquitin ligase BIG BROTHER (BB)  F: AAATGAACGGGAACCAACA  R: GCAGGTGGATGTGTAAGACC |
| > c50934_g1 Transcription factor BPE (BPE)  F: CATTAAGAGGCCATGCTTCC  R: ACAGAAACCAAAGTTCCATGC |
| > c3980_g1 Zinc finger protein JAGGED (JAG)  F: GCAACTCACCTCCTCTCTCAA  R: TGCTCCCATTGTTACTGTGC |
| > c23929_g1 AP2-like ethylene-responsive transcription factor AIL5 (AIL5)  F: TGGATTCTTCTCCGAACTGG  R: GCGGTGGGTATTGTGGAA |
| > c43943_g1 Growth-regulating factor 4(GRF4)  F: CTCCACCCAAGTCTCTGACC  R: GCTGCTCTCTCCATTGCTTC |
| > c50931_g2 Auxin-responsive protein IAA7  F: TCCACAAGAGACGATACAGAGC  R: GGAGAGGAGGTTGAGTTTGAGA |
| > c68768_g1 DELLA protein GAI (GAI)  F: ACCCTCCGCAAAGTAGGTC  R: CGGTTCAGCAAGACAATCTG |
| > c35415_g2 Floral homeotic protein AGAMOUS (AG/AG2)  F: ATCTCAGCCATTTGATGCTC  R: TGACCTGCTTCTTGGTAATCTT |
| > c10961_g1 SEPALLATA 1/2 (SEP1/2)  F: TCAAAGGAGGGAACAAATGC  R: GGAAGGCGGTGATAAGGAAT |
| > c33060_g1 MADS-box protein SVP (SVP)  F: ATTCACGGGTCTTGTCTGCT  R: TGCCGATGTTGCTCTTATTG |
| > c38775_g1 MADS-box protein SOC1 (SOC1)  F: GCACTTATTGTCTTCTCTCCAACA  R: TTCTTCAACGGCTCTTTTGC |
| > c10345_g1 MADS-box transcription factor 6 (MADS6)  F: GGGAAGTGACCTTGGAGAAAG  R: TCAGCCGTGGGTAAAGTGTT |
| > c54467_g2 Agamous-like MADS-box protein AGL15 (AGL15)  F: TGTCTGCGTAAAGCCTCGT  R: CAGTGAAAGAGAGAAAGGAGCA |
| > c20024_g1 Floricaula/leafy homolog 1 (FL1/LFY)  F: TGAGAGGCAACGAGAGCAC  R: GACCAAGAAATCACGGCACT |
| > c57679_g2 FT-like protein (FT)  F: GGCCTAACTGTCGGAACAAC  R: CACTGATATTCCAGCCACCA |
| > SAND  F: CTCCAATCAGTTTGCCCTCACA  R: TATCCGTATTGCCACAGCCTTG |
