# Supplementary material for: Transcriptomic Analysis Reveals Mechanisms of Sterile and Fertile Flower Differentiation and Development in Viburnum macrocephalum f. keteleeri
Source: Front Plant Sci. 2017 Mar 1;8:261. doi: 10.3389/fpls.2017.00261 (PMC5331048; doi:10.3389/fpls.2017.00261)
Supplement: Supplementary file 2 [file Table2.DOCX]

**Table S2** Summary of sequence analyses for fertile and sterile flowers in *V*. *macrocephalum* f. *keteleeri*.

| Sample | Raw Reads | Clean reads | Clean bases | Error(%) | Q20(%) | Q30(%) | GC(%) |
| --- | --- | --- | --- | --- | --- | --- | --- |
| S1_1 | 27389554 | 26454021 | 3.31G | 0.03 | 96.81 | 93.59 | 45.87 |
| S1_2 | 27389554 | 26454021 | 3.31G | 0.04 | 94.39 | 89.76 | 45.82 |
| S2_1 | 22886321 | 22231728 | 2.78G | 0.03 | 96.24 | 92.42 | 45.98 |
| S2_2 | 22886321 | 22231728 | 2.78G | 0.04 | 94.95 | 90.38 | 45.97 |
| S3_1 | 22261760 | 20932873 | 2.62G | 0.03 | 97.1 | 94.09 | 46.67 |
| S3_2 | 22261760 | 20932873 | 2.62G | 0.04 | 94.19 | 89.44 | 46.59 |
| F1_1 | 22176099 | 21539321 | 2.69G | 0.03 | 96.22 | 92.4 | 45.96 |
| F1_2 | 22176099 | 21539321 | 2.69G | 0.03 | 95.68 | 91.63 | 45.97 |
| F2_1 | 23749074 | 23034593 | 2.88G | 0.03 | 96.27 | 92.48 | 45.97 |
| F2_2 | 23749074 | 23034593 | 2.88G | 0.03 | 95.34 | 91.05 | 45.96 |
| F3_1 | 24935684 | 23320317 | 2.92G | 0.03 | 97.05 | 94.02 | 46.2 |
| F3_2 | 24935684 | 23320317 | 2.92G | 0.04 | 94.14 | 89.33 | 46.14 |
| S_Summary | 139047790 | 133401018 | 17.42G | 0.04 | 95.79 | 91.84 | 46.11 |
| F_Summary | 139861815 | 134322544 | 16.98G | 0.03 | 95.52 | 91.42 | 46.17 |
| Summary | 278909605 | 267723562 | 34.4G |  |  |  | 46.14 |

S1, S2, S3: three independent biological replicates for sterile flower

F1, F2, F3: three independent biological replicates for fertile flower

S1_1: Reads sequencing of sterile flower from the left.

S1_2: Reads sequencing of sterile flower from the right.

F1_1: Reads sequencing of fertile flower from the left.

F1_2: Reads sequencing of fertile flower from the right.

Q20: The percentage of bases with a Phred value > 20.

Q30: The percentage of bases with a Phred value > 30.
