# Supplementary material for: Transcriptomic Analysis Reveals Mechanisms of Sterile and Fertile Flower Differentiation and Development in Viburnum macrocephalum f. keteleeri
Source: Front Plant Sci. 2017 Mar 1;8:261. doi: 10.3389/fpls.2017.00261 (PMC5331048; doi:10.3389/fpls.2017.00261)
Supplement: Supplementary file 3 [file Table3.DOCX]

**Table S3.** Summary of mapped read numbers in each sample from fertile and sterile flowers.

| Sample name | Total reads | Total mapped |
| --- | --- | --- |
| F1 | 43078642 | 35241432 (81.81%) |
| F2 | 46069186 | 37677028 (81.78%) |
| F3 | 46640634 | 37897940 (81.26%) |
| S1 | 52908042 | 42517730 (80.36%) |
| S2 | 44463456 | 36422084 (81.91%) |
| S3 | 41865746 | 34133686 (81.53%) |
