# Supplementary material for: Transcriptomic Analysis Reveals Mechanisms of Sterile and Fertile Flower Differentiation and Development in Viburnum macrocephalum f. keteleeri
Source: Front Plant Sci. 2017 Mar 1;8:261. doi: 10.3389/fpls.2017.00261 (PMC5331048; doi:10.3389/fpls.2017.00261)

Figure S1. Annotation and function classification of the assembled *V. macrocephalum* f. *keteleeri* unigenes.

A

Database	Number of Unigenes	Percentage (%)
NR	38224	36.16
NT	20724	19.6
KO	12201	11.54
SwissProt	26744	25.3
PFAM	26629	25.19
GO	30381	28.74
KOG	13637	12.9
Total Unigenes	105683	100

B

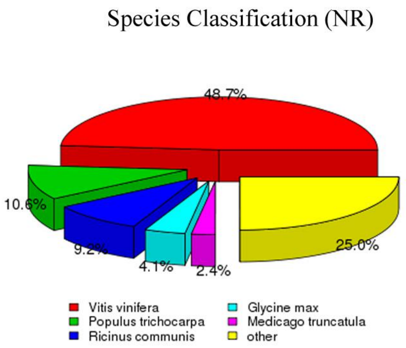

C

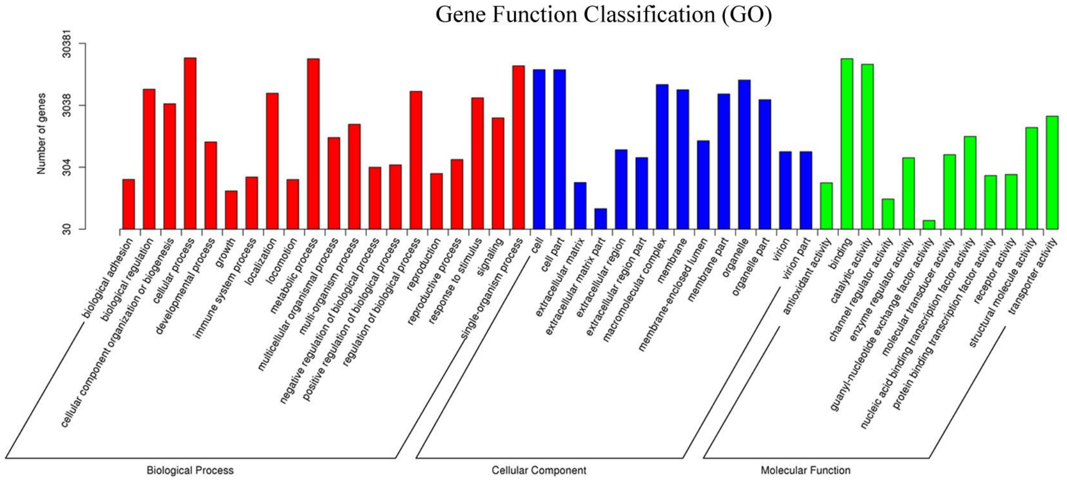

D

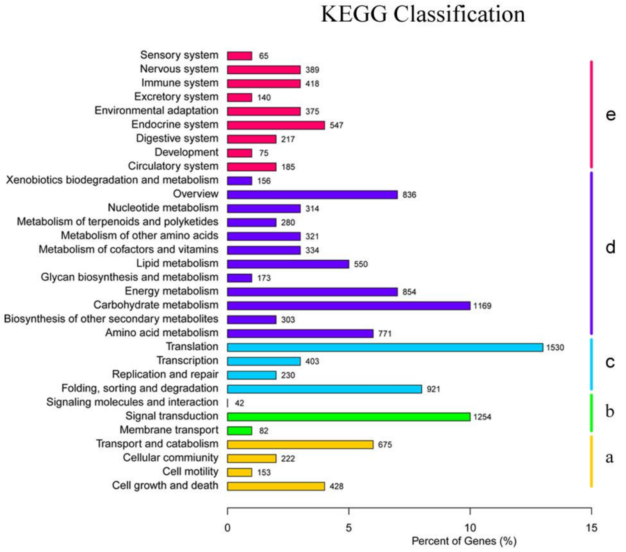

Supplement: Supplementary file 11 [file Image1.PDF]
